# Young Women's Experiences of Violence and Homelessness

**DOI:** 10.1177/10778012241243053

**Published:** 2024-04-13

**Authors:** Sarah Wendt, Kristin Natalier, Sharyn Goudie

**Affiliations:** 1 104528 Department of Social Work, The University of Melbourne, Melbourne, Victoria, Australia; 2 College of Education, Psychology and Social Work, 1065 Flinders University, Adelaide, South Australia, Australia; 3 College of Humanities, Arts and Social Sciences, 1065 Flinders University, Adelaide, South Australia, Australia

**Keywords:** domestic and family violence, homelessness, young women, trauma, service response

## Abstract

This article explores the lived experience of young women navigating and surviving domestic and family violence (DFV) and homelessness. Promoting the voices of young women through in-depth interviews, this article considers their story of violence, abuse, homelessness, and sense of safety. Such stories enable reflection on the ability of services to provide personal, material, emotional, and cultural safety in a way that recognizes the intersecting impact of trauma before, during, and after experiences of DFV. We conclude by arguing that providing emotional and cultural safety through the relational aspects of service delivery is essential to developing a strong foundation for young women's futures.

## Introduction

Domestic and family violence (DFV) is a leading cause of women's homelessness across their life course—with various complexities and experiences in different age groups (Baker et al., 2010; Franzway et al., 2019). Experiences of DFV in childhood increases young women's vulnerability to homelessness. Through the erosion of a sense of safety and belonging it can become necessary for young women to leave home to escape the violence (Chamberlain & Johnson, 2013; O’Campo et al., 2016; Zufferey et al., 2016). In the context of DFV experienced by young women, financial abuse and social isolation typically co-occur with physical violence. These factors can further exacerbate the risk of homelessness and housing instability by limiting the economic and social resources that might otherwise assist women to attain sustainable housing (Broll & Huey, 2020; Sanders, 2015). Violence can also erode family networks as sources of emotional, housing, and financial support that might otherwise limit periods of homelessness (Groton & Radey, 2019; Natalier & Johnson, 2012). Thus, homelessness reflects complex interactions of structural and biographical factors, rather than a single cause or identifiable event sparking a trajectory into homelessness.

Phipps et al. (2019, p. 7) argue that understanding how women come to homelessness is a beneficial starting point to potentially identify earlier intervention and social services responses. However, this understanding can only be achieved through a deep analysis and naming of the complex issues of these women's lives. It requires an opportunity for young women to tell their story of homelessness, violence, and abuse. Responding to Phipps et al.'s (2019) call, this article explores the lived experience of 22 young Australian women navigating and surviving DFV and homelessness across their lifetime. It provides an account of the complex relationship between young women's experiences of interpersonal violence, housing insecurity, and sense of safety. The findings highlight the importance of relationships in informing survivors’ experiences of safe and unsafe housing and the services that can best support them. Through this focus, the article sheds light on the capacity for homelessness services to provide the young women with the safety they are seeking. In doing so, the article contributes to understanding the ways in which young women's experiences of violence and safety shape their housing and provides insight into how service supports that are sensitive to lived experiences may be used to increase women's sense of safety.

## Literature

In this article, homelessness here is understood as a continuum of primary, secondary, tertiary, and marginal levels of homelessness; hence includes rooflessness, couch surfing, temporary shelters (including supported accommodation), inappropriate or insecure residence, and those at risk (Zufferey, 2016). The relationship between DFV and homelessness and housing insecurity for young women is shaped by challenges including childhood trauma, poverty, a lack of safe and secure housing options, greater vulnerability for further experiences of sexual violence and DFV, and the need for appropriate support for those exiting homelessness. Each of these issues intersects based on age, race, ethnicity, gender and sexual identity, and disability.

Childhood trauma is a key theme in young women's accounts of trajectories into homelessness. For young women, homelessness often reflects cumulative risk and adversity associated with an abused and fragmented childhood and adulthood and a lifetime exposure to victimization and trauma (Broll & Huey, 2020; Cronley et al., 2020; Phipps et al., 2019; Warburton et al., 2022). Thus, women who are subject to multiple forms of victimization across their life course are more likely to experience more than one episode of homelessness (Broll & Huey, 2020).

DFV is also associated with women's lack of financial resources and unemployment or underemployment both directly and indirectly. Directly, through financial abuse (whereby abusers control women's access to money, including preventing them from engaging in paid work), and indirectly, through the social isolation and physical and mental impacts of DFV that can limit women's ability to engage in paid work (Baker et al., 2009; Tually et al., 2009). Furthermore, the need to flee violent environments at short notice often leaves young women with minimal assets or the time to secure future housing (Warburton et al., 2022). Due to these factors, when young women leave home due to violence and are reliant on social security payments, they face longer-term poverty and challenges in attaining and maintaining housing (Johnson et al., 2008; Tually et al., 2009).

DFV support and crisis housing support cannot address the constraints of the housing market faced by these young women (Blunden & Flanagan, 2022; Flanagan et al., 2019). Challenges include a lack of affordable and appropriate housing stock across social housing and private markets, along with landlord discrimination (because of age and ethnicity). This lack of housing can mean that young women feel they have little choice but to accept accommodation that is inappropriate, unsafe, or far away from informal support networks and formal supports (Blunden & Flanagan, 2022; Flanagan et al., 2019). These barriers to achieving stable housing may increase young women's risks of ongoing or additional episodes of homelessness (Blunden & Flanagan, 2022; Tually et al., 2009; Warburton et al., 2022).

Homelessness can also further increase women's vulnerability to sexual assault and DFV (Murray, 2011). Unsafe crisis accommodation, rough sleeping, and couch surfing can leave women and their families vulnerable to sexual and physical abuse (Barnes et al., 2021; Murray, 2011). Sexual violence is widely perpetrated against homeless women (Robinson, 2011). Furthermore, when homeless, young women may be dependent on a violent intimate partner for shelter, money, and emotional connection (Cornell-March & Sandstrom, 2015). Homeless young women may experience reproductive coercion and exercise little control over their sexual and reproductive lives (Cronley et al., 2020). Transactional sex (Watson, 2016), moving between homelessness and violent homes (Chamberlain & Johnson, 2013), and exploitative social relationships (Barker, 2014) have all been identified as behaviors that are simultaneously survival strategies, may contribute to a subjective sense of safety, and increase young women's risk of being a victim of violence. Thus, DFV and sexual assault are not only causes of homelessness, through the compounding trauma, they can extend or complicate young women's homelessness (Broll & Huey, 2020; Cronley et al., 2020).

Mayock et al. (2015) concluded that attempting to exit homelessness without support from services or family may contribute to ongoing homelessness. In particular, employment, education, and training supports are needed to strengthen women's ability to recover from the impacts of DFV and/or sexual assault and retain sustainable and appropriate housing (Klein et al., 2021). Exiting homelessness requires more than simply housing but also income support and/or employment, health provision, family, and community support and often case management (Schwan et al., 2019), Currently, young women escaping DFV are often bereft of these supports.

Recognizing that the relationship between homeless and DFV is an ongoing process, Baker et al. (2003, p. 776) suggest that the relationship between DFV and housing needs to be reconceptualized, “from one of leaving, to one of gaining safety.” Safety in this article is understood to be multidimensional, contingent and experienced rather than objectively measured. This reflects women's desire for connectedness, stability, security, and safety for themselves and their children (Biederman & Forlan, 2016; Kirkman et al., 2015). In the case of women who have left DFV relationships, the literature identifies three dimensions of housing-related safety. These are personal safety (Benbow et al., 2019; Fraga Rizo et al., 2022; Zufferey et al., 2016), material safety (e.g., physically safe housing, safe surrounds; Benbow et al., 2019; Clough et al., 2014; Fraga Rizo et al., 2022; Hetling et al., 2018; Kirkman et al., 2015; Zufferey et al., 2016), and emotional and psychological safety (Fraga Rizo et al., 2022; Hetling et al., 2018; Woodhall-Melnik et al., 2017). Cultural safety is also important in creating safe housing (Christensen, 2016; Fang et al., 2023). These multiple dimensions of safety highlight the complexity of Sullivan et al.'s (2019, p. 199) comment that through the “process of locating and obtaining housing, advocates and survivors always need to keep safety in mind.” This complexity is intensified by the pressures on service providers, including funder requirements, service demand, institutional logic, and socio-economic structures (Cattaneo et al., 2021).

## Method

The aim of the research design was to let young women speak for themselves and tell their story of violence, abuse, homelessness, and sense of safety. In doing so, the study sought to generate data to identify key patterns in young women's needs and service use to inform best practice in service provision to young women. Using a qualitative design, this article addresses the following research questions:

1. What is the relationship between DFV and sexual assault and homelessness and insecure housing?
2. How can access to stable housing be used to increase women's sense of safety?

### Conceptual Framework

The study was informed by intersectional feminist ideas for two reasons. First, it acknowledges home, and homelessness are embodied and gendered, which centers the main driver of women's homelessness in the context of unequal gender and other social power relations (Zufferey, 2016). Second, it draws attention to diversity and enables research into DFV and sexual assault to identify women's experiences in relation not only to gender inequality but also to other social divisions such as class, race/ethnicity, age, sexuality, and disability (Cavanagh et al., 2013). Intersectionality captures the experiences of women who find themselves marginalized from dominant framings of homelessness and violence and identify with other forms of oppression beyond gender (Cavanagh et al., 2013). The research project therefore paid attention to recruiting Aboriginal and Torres Strait Islander, and culturally and linguistically diverse women, to the study and drawing out their experiences and needs. Through capturing the voices of young women talking about safety and how their understanding of safety link to housing/home, the research offers an intersectional representation of age, mothering, ethnicity, and race together with experiences of service provision.

### Recruitment and Sample

Young women were recruited using two strategies. First, 13 women aged between 18 and 30 years were recruited through six service providers in metropolitan Adelaide, South Australia, working with young women because of violence and safety concerns and/or housing concerns. The young women were given the chance to discuss the research with workers before committing to participating in an interview, so that they could explore questions and concerns and had time to meaningfully consider whether they wanted to be involved.

Culturally and linguistically diverse and Aboriginal young women were recruited by working alongside Aboriginal local organizations and migrant health agencies. Agencies that offered supported accommodation were particularly helpful in enabling young women to participate. Supported accommodation in this context represented agencies that provided individually fully furnished family units and support including setting personal goals, education, training, and finding and maintaining appropriate long-term housing.

An additional nine young women were recruited directly via targeted Facebook advertising. These young women had experienced violence or housing instability but were not engaged in service systems. The advertisement was viewed a total of 38,399 times, with the “learn more” link in the advertisement being clicked on 168 times. A total of nine young women registered their contact details—and all of these nine women then went on to participate in an interview at Flinders University's campus.

Before the interview, the researcher spoke with participants about the purpose of the study and explained to the young women their right to withdraw at any stage without penalty, and guaranteed confidentiality and anonymity. All of the young women were provided with the option of having a support person at the interview, however, only one young woman opted to have her support worker present. The researchers also put in place strategies and assistance for all young women to access counseling/support to debrief after an interview (or at any point during their participation in the research process) where this was required. Only one woman sought additional support from her worker after the interview. In line with common research practice, an honorarium of $50 was provided to all young women participating in the interview process, to acknowledge their contribution to the project. This was provided as a gift card. Participant privacy and confidentiality were maintained throughout the entire process and pseudonyms have been employed for all participants to maintain anonymity. As Table 1 shows, the final sample included 22 women aged between 17 and 38 years. Twelve women had children, two women had current intervention orders, and all women but two had separated from their partner.

**Table 1.** Sample Characteristics.

Pseudonym	Age range (years)	Children	Partner	Ethnicity	Employment or education/training
Catherine	18–24	1 child aged under 5 years	Intervention order	Anglo-Australian	Unemployed
Chloe	18–24	1 child aged under 5 years; 1 child aged over 5 years	Two separate fathers but not living in Australia—no contact at all	Black African	Unemployed
Carol	18–24	1 child aged under 5 years	Intervention order	Anglo-Australian	Unemployed
Charlotte	18–24	1 child aged under 5 years	Separated—some contact	Anglo-Australian	Unemployed
Jade	18–24	1 child aged under 5 years	Separated—some contact	Anglo-Australian	Unemployed
Michelle	18–24	1 child aged under 5 years	No contact at all	Anglo-Australian	Unemployed
Margaret	18–24	1 child aged under 5 years	Separated—some contact	Black African	Unemployed
Melissa	18–24	1 child aged under 5 years	No contact at all	Aboriginal	Unemployed
Melanie	18–24	1 child aged under 5 years	Together	Anglo-Australian	Unemployed
Helen	18–24	1 child aged under 5 years; 1 child aged over 5 years	No contact at all	Aboriginal	Unemployed
Harriet	15–17	No children	N/A	Aboriginal	Education
Yvonne	18–24	No children	N/A	Aboriginal	Unemployed
Abbie	15–17	No children	Cohabiting	Anglo-Australian	Education
Fiona	18–24	No children	N/A	Anglo-Australian	Education
Felicity	18–24	No children	N/A	South Asian	Education
Fay	Over 30	1 child aged over 15 years	No contact at all	Anglo-Australian	Unemployed
Francis	25–29	1 child aged under 5 years; 1 child over 15 years	Separated—some contact	Anglo-Australian	Unemployed
Gabby	25–29	No children	Separated—no contact	Anglo-Australian	Education
Gloria	25–29	No children	Separated—no contact	Anglo-Australian	Education
Ebony	18–24	No children	N/A	Middle Eastern	Education
Edel	18–24	No children	N/A	Anglo-Australian	Education
Ella	Over 30	1 child aged over 5 years	Separated—no contact	Anglo-Australian	Education

### Interviews

In-depth, semi-structured interviews with the 22 young women who had experienced violence and homelessness explored the nuances and multidimensionality of young women's lived experiences, the relationships between violence, housing/homelessness and safety, and their support needs. The interview guide was developed in consultation with a Stakeholder Advisory Group and addressed the following themes: demographics; housing trajectory and perceptions of appropriate and safe housing; experiences with DFV and sexual assault; relationship between housing and DFV and sexual assault; use of services; and perceptions and experiences of different engagement strategies. Interviews lasted between 30 min and 1 h and all were audio-recorded and transcribed by a reputable transcription service.

### Analysis

The analysis of young women's interviews was guided by narrative analysis, an approach that emphasizes the story-based nature of human understanding. There were three layers of analysis that allowed the researchers to focus on each young woman's story as it incorporated themes of safety, home, and experiences of violence. First, by keeping the story intact the researchers were then able to compare each young woman's experiences with those of others in the sample. Second, three specific groups were then identified: women with children receiving services, women without children receiving services, and women who had not accessed services. To further comparison, thematic analysis was then used to identify commonalities and differences within the three groups of women. As a final step, thematic analysis was used to identify common themes across the whole sample. The study was approved by the Human Research Ethics Committee, at Flinders University, Project ID: 8201.

## Findings

The findings for each group of women are discussed below, sharing experiences of violence that contributed to homelessness. The experiences of service responses are also outlined and the women's understandings and reflections on safety.

### Young Women With Children Accessing Services

Among the young women who were interviewed for this research, there were 10 young women with children who were being supported by a service at the time of the interview. All were accessing a DFV-supported accommodation service specifically for young women. They were eligible for the service because they were pregnant and/or had children and had experienced DFV—and hence were at risk of homelessness.

Six of the 10 young women talked about their childhoods as being unsafe and frightening because they experienced violence from their mother, father, or stepfather. They also spoke about feeling alone, neglected and just trying to survive long enough to be able to leave. For some women, leaving home was associated with new experiences of violence, with sexual assault perpetrated by either teenage peers, employers, their mother's partners, or fathers. Most young women who left home experienced housing instability, staying with friends, extended family, or grandparents for short periods of time until they secured support from a youth service or found a boyfriend they could live with. Catherine described such moments in her life:

He [stepfather] punched me in the face just for crying … I hated him, didn’t feel safe around him. And then we moved again when I was about 13 years old, everything went downhill from there, everything. I got so severely depressed, my mental health went downhill … and then when I was 15—I was hurt really bad by a group of boys and I got worse … but then I met a boyfriend and I was with him for a couple of years, I moved in with him straight away, got out the house. I was fifteen, moved out.

When the young women described moments of transition, such as moving between their mother's or father's homes, between friends, or moving in and out of youth shelters, they often linked these transitions to experiencing sexual assault. For example, Jade said:

And I moved in with his friend, my Dad's family friends. And living there, and I’m getting sexually assaulted, so I just stopped trusting anyone, and just started being homeless, because it's like everywhere I go, because they kicked me out, or I’d be screwed over.

For some women, youth services were helpful for short periods of time, but they often moved out of the youth service because they partnered with a boyfriend. For example, Carol said:

I was 15 and couch surfing until I got into [youth service]. I was there for about six months. I went down to Centrelink, got on a payment and it just all started from there. There was no going back for me, I wasn’t going back to that sort of house [her family home]. But then again, I also spiralled, I started smoking and smoking more, I was hanging out with the wrong crowd. I was then put into a youth share house and I was in and out of there for a year until I kind of just started pulling my head out … but then I found [partner] and fell pregnant.

It was common for the young women to find housing with their partners either through living with their partner's family or securing a rental or community house as a couple. It was also common for young couples to become pregnant. Many of the young women described DFV early on in their relationships, beginning or intensifying when they were pregnant. They then felt trapped and had nowhere to go because they were dependent on their partner and his family and had no independent finances to support themselves and their baby. Jade described this fear:

I got transferred to [suburb—independent housing through a youth service], and that is where I met my son's father. And at the time, we were on ice and I fell pregnant, and I had my [child] while I was living at that house, and I’d just turned 18. I wasn’t contacting my worker or nothing, they just gave me an eviction notice, and my [child] was like, one month old or something like that … we managed to sub-lease but there was a big fight with my partner, and it was around the baby and that, so I called an ambulance, just to make sure that he was okay. So, we went to hospital for three days … He’d been sent to jail. I couldn’t afford the rent. The rent was like all of my money, so there was no food or nothing like that … And then I made calls to all the services and that, and there was nothing available, and it was getting to like the point where I was so scared, then [DFV service] called.

Four of the 10 young women did not describe violence and neglect in their childhood. Their discussions of violence centered on the violence perpetrated by their partners and fathers of their children. This violence led to an erosion of family or social networks, so that women had no informal supports when they wanted to leave the violence (primarily because of their children). For example, Chloe was a child refugee from Africa. She moved to Adelaide with her daughters but the acquaintance who was going to offer her accommodation withdrew the offer; hence she rang service after service looking for accommodation. Similarly, Helen, an Aboriginal woman, described periods of living with her ex-partner's family—and when she became pregnant to another man, she was kicked out of this accommodation. She experienced a period of unstable housing with her child and when she had her second baby accommodation was arranged for her through the DFV service sector. For Melissa and Margaret, it was an abusive partner that caused them to access the DFV service sector because their extended family did not have the capacity to support them. For example, Melissa, an Aboriginal woman, said:

Me and [my child's] dad, we split when I was four months pregnant and I haven’t really spoken to him since. It was really at the start and then drugs got involved and yeah, he just went off the rails. Ended up going to prison and yeah. And he's just only gotten out so, yeah, I haven’t seen him … because he's got children to different women, two other women. Yeah, it just was really hard for me to try and be a step-mum to them—and then deal with the mothers on top. And then they was pissed off with him for getting me pregnant and him not being there for his other children … I was just always arguing with my parents, so I couldn’t live with them and I had really nowhere else to go … so I contacted [DFV service], had an appointment and then five days later they contacted me saying I could move in.

In this sub-sample, all of the women were living in supported accommodation. This provided an opportunity to talk to the young women about their experiences of such a service response. At the time of the interview, the women were either living in onsite accommodation or had been transferred to an outreach medium- or long-term property. All of the women spoke positively about this service response. For some women for whom safety was a significant concern, the service gave them security and time to think about their options. For example, Catherine said:

I stayed on site for a few months, probably three months or something and then I went to the outreach house. I was so relieved and that's what's so crazy … I had such a sense of relief. I was scared to be by myself at night though, but I had such a sense of relief, like I was finally free, I was … I wasn’t sad, I wasn’t—I didn’t cry and stuff like that, I didn’t—I was just alright.

All of the women spoke positively about the workers without prompting. All noted that they never felt judged, only supported—in terms of being a mother, through practical material possessions and through counseling and emotional support. Michelle described the workers:

The workers are really lovely, I have had problems in the past with dealing with judgy workers. But here they are not, they’re all understanding, they’re also practical. So even if you’re—I don’t know how to explain this. So practical in a way we have parenting groups, cooking classes, and that is a good way to talk to other young mothers.

Margaret particularly valued the support of workers. In Margaret's culture there are practices that are important to women after they have a baby. She explained these practices to the workers and the workers facilitated her sister to stay with her in the accommodation so that Margaret could undertake these practices:

I told them how I was pregnant and normally my traditional thing like not to be in the kitchen for a month and they allowed my sister to stay with me … I couldn’t do this without my sister, and my sister, because she doesn’t drive, so by now she stays with me here and I am so, I was so panicked and now my sister is here with me. Now I am happy, I don’t stress—like before I used to cry all the time and I am like, when I am crying I wouldn’t even have a reason why I am crying, but here, since I came I don’t cry.

All of the women in this sub-sample spoke about how the DFV service and the associated housing gave them a sense of safety, both materially and emotionally. For young women like Catherine, who identified as having severe anxiety and depression because of the violence and instability she has endured, supported accommodation offered her a sense of safety that had previously been absent from her life:

Having a safe place to come through all this, especially living for 21 years feeling unsafe everywhere I’ve been and moved around left, right and centre. And for the past year I’ve actually felt safe for the first time in my life … I just want to stay here and know that there's cameras here, there's someone on site, it's behind gates … I get scared walking to my car at night.

For other young women, housing offered the opportunity to pursue school, study, or other activities. For example, Chloe described how the stability of housing enabled her to enroll her children into child care, get her driver's licence, and finish her Year 12 studies:

I go to different house inspections and was getting worried, but the worker, she helped me with the paperwork and took me see houses and kept telling me to not worry: “Be patient and we will try to help you some way, you just need to calm down.” … So, I took her advice and stuff … she even promised me that if they can’t find a place for me they won’t just ask me to leave … Make sure we find a place, everything we needed. And that's exactly what I have now. I got my licence, they also helped me to put the [children] in school and child care as well. My main focus now is to finish my Year 12.

For many of the young women, supported accommodation created a sense of emotional safety, breathing space, a sense of calm. Melissa, an Aboriginal woman, shared how housing provided her the opportunity to reconnect with her own mother. Melissa's partner was sent to jail and she did not have contact with him. She loved her family but she couldn’t live with them because they struggled with their own drug and alcohol problems. She didn’t feel safe to have her baby while living with her parents:

I guess with all the support workers here and just being highly surveillance-d and yeah. Just feels homey. It's really good having my own space, not being told what to do, when to do it, who I can have over and yeah, just, I feel independent now. Just basically being able to do things on my own without Mum telling me how to do it. I also spoke to her about if you’re going to be abusing drugs I don’t want something like that and then it's going to jeopardise me with him [her child] and I just can’t even have that … Well, my lease is for three months. I’m hoping I get either community housing or a private rental. I’m not really fussed, either, I definitely still want my own housing.

Housing support and willingness of the agency to enable women to navigate and mend relationships with their families (particularly their mothers and sisters) were particularly valued. Maintaining connection to their culture and families helped them feel hopeful while also allowing them to take steps toward their independence as young mothers.

### Young Women Without Children Accessing Services

There were three women who did not have children and were receiving a youth service because of homelessness risk or a sexual assault service because of a recent experience. All three young women in this sub-sample described violence and neglect in their childhoods, which caused them to leave home. Yvonne was removed from her family through child protection processes and experienced housing and emotional instability as she lived with multiple foster families. Harriet and Abbie experienced periods of transience and living with friends and boyfriends until they were supported to access a youth service for accommodation. In the case of Harriet:

She [mother] kicked me out, I didn’t like her new boyfriend, I was too much to handle apparently. Then I was homeless for a little bit there, like staying at a football oval … couch surfing at me mates’ and whoever else took me in. And then I spoke to my counsellor in high school. They did all they could and put me in contact with [youth service].

Harriet, Yvonne, and Abbie all accessed supported accommodation through youth services. It is worth sharing their experiences one by one, to offer insights into opportunities gained and lost in that service provision. For Harriet, the youth service offered stability and routine. She was able to continue with school. She found the workers available and supportive. Supported accommodation was vital to her as it enabled her to gain and plan for independence:

You pay your rent, you do your own thing—but they’re just there to help. There's other people that live in the building; like other tenants that live in the building, but that your space is your space. … When I was homeless, I was always down, I started smoking weed and stuff, like that's just not me. So, like I’m glad like I got into this place. I think it's just more the fact that I am actually stable.

Yvonne lived in state care from 8 years of age. She spoke about how she wanted to leave her supported group housing when she turned 18; however, she was given no choice about the type of housing—although she did have a say in the suburb. She struggled with the emotional and practical demands of living independently, with no meaningful assistance from support workers:

It was hard. I was just, depended on my carers just being there and then when I moved to the new house I just couldn’t—like, I’m used to somebody there with me in the house. Like just, just hanging out with me—because I like company and if I’m by myself I sit there being bored.

Yvonne stayed in her initial house but felt unsafe. Her neighbors yelled, screamed, and fought—and so the police were called frequently. Yvonne was the only woman in the unit block. Her neighbors were drug dealers and there were people coming and going at all hours of the day and night. Yvonne endured this for just under 2 years before she was moved to another property. Similarly, Abbie lived in supported accommodation with other young people. She often felt like she didn’t belong, and she moved in and out of this for periods of time to be with her boyfriend. When Abbie turned 18, she transitioned out into an independent living unit, where she felt unsafe. Like Yvonne, she was placed in a block of units where her neighbors’ behavior increasingly made her feel unsafe:

My unit got broken into, and I was assaulted. Yeah, I’d pretty much just given up and it doesn’t become a home or really a house to me even. It just feels like a dump because people can treat it like a dump. People can treat me like a dump. I am now extremely scared to be on my own.

The three young women reported that the youth service-supported accommodation was pivotal in ensuring their physical safety and helping to improve their well-being. However, all three young women talked about struggling with mental health issues, and hence safety was not a sustained feeling for them. The secure housing reassured them physically, but they described feeling emotionally unsafe. For example, Yvonne said:

I was referred to my doctor because I was—with my mental health state, I was injuring myself a lot, like I was in depression, really bad depression that I always end up trying to kill myself but this place has helped me through it.

### Young Women Who Were Not Accessing Services

Nine young women were recruited through Facebook and were not accessing housing or DFV/sexual assault services at the time of the interview. These women discussed DFV being present in their childhoods or experiencing intimate partner violence as a young woman or both. They navigated housing options largely on their own or had friends or family who helped with housing.

Six of the women in this sub-sample described DFV perpetrated by their fathers against their mothers, themselves, and their siblings. The violence was severe, but the young women remembered that it was “normal,” that is, what fathers do. It was only once they approached their teenage years that they began to plan to leave home and leave the violence. Some women also described their struggles with mental illness growing up as a result of the DFV and sexual assault-related trauma they endured, particularly in their teenage years. Some also linked the trauma of DFV to their mother's mental illness and believed the violence contributed to their mother's inability to recover longer term. Edel spoke about her own mental health suffering because of the DFV she endured throughout her childhood:

There was a few times I remember where I was—5 or 6 and I’d walk out and my Dad was on top of my Mum while she was laying there and blood around her head and stuff and then my Dad would just leave the house and I’d be there to help Mum pick everything up afterwards … my Mum would leave with us and return, time and time again … I struggle with paranoid schizophrenia, but I was untreated for some time … all the violence and anger around helped me and my sister and my Mum be close—form like a bond even though we were—I—that was the point where I was hitting puberty and had mood swings and we’d always be fighting but—I always knew that there was …

Two women spoke about experiencing intimate partner violence as young women and their parents being a source of support, helping them to find safe and stable accommodation once they left the relationship. Both women discussed DFV becoming increasingly severe and controlling once they were pregnant.

At the time of the interview, the women recruited through Facebook were not receiving services for DFV/sexual assault or housing support. Some were seeing a private psychologist to assist them to understand their own trauma and to heal from childhood experiences and intimate partner relationships. The young women were able to provide reflections on the services they did receive in the past. Fiona, Fay, and Gabby talked about their memories and experiences of the Family Court. They remember it as a service response that took them from their mothers and enabled their fathers to continue abusing them. The young women talked about leaving home and therefore their fathers as soon as they were old enough and relayed their sadness at losing their mothers and contact with their siblings as they got older. For example, Fiona's story captures this similarity across the lives. She said:

I remember the crèche area, but it was designed for 3–4-year-olds and I was older, and so I was really out of my element and uncomfortable. I remember we had to talk to all these complete strangers about things I didn’t even understand. We had to go into a room, like a viewing room … we went there for two weeks straight, Monday to Friday, I just remember Mum sobbing her eyes out, walking in and it all being very traumatic and just crying all the time, it was just awful. And so I’m not even sure how many times they went to court … I just remember pretty much Mum dropping us off one day and Dad picking us up and I didn’t see Mum for six months after that.

Fiona spoke about sexual abuse from her father during this time and described vivid memories of rape. Numerous times she ran away and went to her mother's only to be returned to her father because of the Family Court ruling. However, by the age of 13–14, Fiona said she just continued to go to her mother's and then ended up not seeing her father for 2 years. Fiona also described her mother suffering from mental illness and how she as a young woman saw this as trauma-related. She described her mother as being “worn down” by the system and her father. At the age of 16 years old, she moved in with a friend and experienced stability for 2–3 years while she finished school. Fiona spoke of receiving no support services, only visiting a police station with her friend to report the sexual abuse by her father.

Francis, Felicity, and Ebony talked about receiving a good service response from police and a specialist domestic violence agency. Francis also had the support of her family throughout this experience. Francis planned to leave her violent partner and worked with a specialist domestic violence agency to do this. Felicity and Ebony spoke about the violence perpetrated by their fathers. A culturally sensitive and quick crisis response enabled their safety. For example, Felicity spoke highly of the police and the referral and support to a domestic violence agency that offered support to her mother after her father broke the intervention order they had in place. Similarly, Ebony received support from both police and a specialist domestic violence agency when her father breached an intervention order:

The police officers that responded were good … they called the Domestic Violence Crisis Association; something like that. And they were helpful, because they helped me with the whole legal side of it and provided, like, support if I ever wanted to talk … so, I’d feel more safer going to court. So, yeah. They were really nice about it.

The remaining three women, Gloria, Edel, and Ella, did not experience extensive service support. Gloria talked about the importance to her of employment and education so that she had financial independence. Studying at university enabled her to leave home. When she experienced DFV with her first boyfriend, she saved money and left. Gloria remembers ringing a domestic violence support agency to talk through her options, but said she was not eligible for crisis accommodation because she had income and could move back with her parents. Gloria sought out counseling, which she found helpful. Edel was seeing a psychologist because of her mental health concerns but said she could move back in with her mother after her experience of independent living did not work out. Finally, Ella had financial and housing support from her parents to leave her violent partner. Ella said she received counseling for a period of time.

When the young women in this sub-sample were asked to reflect on their feelings of safety they mostly reflected on their childhoods where they felt unsafe. They explained that hence as young women they try to create spaces of calmness in their lives. They also described that they don’t trust people very easily and feel uneasy in their lives. For example, despite having safe, secure housing, Gabby talked about a relationship with a boyfriend that had ended and how she felt unsure and unsafe in that relationship. She spoke about the unease she feels in terms of not wanting to make the mistakes in her relationships that would result in repeating experiences from her childhood:

I grew up in an abusive home in my younger years and so, again, I think that made it really difficult just recognising that there was a problem and not wanting to repeat stuff that happened to me previously in my life, I think just made it a lot harder … I didn’t want to be back in that situation again, I didn’t want to admit that I was in a situation that was unhealthy and … was not safe … so this worries me.

The young women who managed to secure a house on their own discussed the joy they felt and the security it provided them. For example, Fiona talked about securing a private rental and going to university. She said:

But definitely when I first moved into that house by myself, I reckon that was in the uni holidays and I just worked so hard to make that house mine. I was doing renovations myself and gardening and everything and spending way too much money on home décor and everything, everything was perfect. And I think I did that to make it my own, make it my own space. And now I have so much stuff now where I am now pretty much your friends, we all have similar ideas and we’ll keep it organised and clean and all homely, have blankets and everything all cosy so. No I think at that point nothing could scare me, I’d seen it all. And I was just confident enough to deal with whatever got thrown my way.

## Discussion

The young women participating in this research shared experiences of violence that support existing findings in the literature. Their experiences again show that DFV is a key driver of women's homelessness (Australian Institute of Health and Welfare, 2018; Franzway et al., 2019) and housing instability, over the immediate and longer term (Baker et al., 2010). Like other studies, this research found that homelessness contributes to women's vulnerability to sexual assault and violent and exploitative relationships (Cornell-March & Sandstrom, 2015; Murray, 2011; Robinson, 2011). Thus, responses to DFV and homelessness and housing instability need to address the cumulative risk and adversity experienced by young women across their life course (Cronley et al., 2020).

Past research has also highlighted the widespread presence of childhood trauma, fragmented family and wider social relationships, and lifetime exposure to violence as direct victims and witnesses (Broll & Huey, 2020; Phipps et al., 2019, Warburton et al., 2022). The majority of the young women described either childhood abuse and neglect in the context of parents’ drug and alcohol use/addiction, mental health issues, or DFV. The perpetrators of physical violence included mothers, fathers, older siblings, and mothers’ and fathers’ new partners and friends. The perpetrators of sexual violence included fathers, stepfathers, and fathers’ friends. For some young women, this violence co-existed with DFV perpetrated by their father against their mothers.

As children, these young women had somewhere to live but felt scared and trapped. Their accounts highlighted experiences of DFV that eroded personal and emotional safety and, in some instances, the material safety of their surroundings. They were, in Wardhaugh's (1999) phrase, “homeless at home”—that is, they did not experience the safety, stability, control, and supportive relationships that give home its meaning and significance beyond a physical dwelling (Mallett, 2004). In response to DFV and trauma, the young women sought to leave their family home as soon as they could—some maintained a relationship with their families, others did not. This leaving was typically associated with unstable, inappropriate, or unsafe housing and experiences that intensified existing trauma. All of these women experienced a violent relationship with their intimate partner once they had left home.

A smaller number of young women first experienced DFV with an intimate partner and eventually left their accommodation for their own and their child's safety. Regardless of when the young women first experienced DFV, this violence contributed to their often continuing fear of transience, instability, and loneliness. The young women felt uncared for, unwanted, and disregarded in their key relationships and in their place in the world. Their immediate experiences of an absence of physical and personal safety extended beyond the initial violent relationship, across their life.

Relationships were also centrally important in shaping useful services. For the young mothers, a service response that offered a supported, secure, and respectful environment allowed them to escape violence and abuse and have their baby or raise their children—and this was vital to contributing to their sense of safety and autonomy. These needs echo the work of Watson and Cuervo (2017), who argue that recognition of the complexity and uniqueness of homelessness trajectories can erode the alienating “us” and “them” service provision experienced by homeless young people. Citing Young's (2001) work, they argue for autonomy, self-respect, and empowerment as necessary in any support for homeless young people. In the current study, support was most effective when it respected and supported mothers’ cultural practices, assisting women to feel connected to their culture and important people from that culture. This offered a sense of emotional safety. In secure housing, this emotional safety intersected with physical and material safety to enable the young women to move from making decisions in a crisis, in a highly anxious state, to a state of greater calm and consideration. This allowed them time to get to know their baby, strengthen relationships with their children, and learn about being a mother—a role they highly valued.

Young women presented divergent interpretations of housing. Some said the housing—which physically separated them from their wider social networks while still allowing visitors on site—gave them a sense of safety, freedom, and autonomy; whereas for others it also brought with it loneliness or a sense of constrained independence (Kuskoff & Mallett, 2016), reflecting a tension between emotional and material safety. Existing research has found that young homeless women often feel lonely, isolated and excluded from meaningful family and peer relationships and from normative social practices and life trajectories. These feelings extend to their relationships with their service providers and it is important for service providers to be aware that many marginalized young women are sensitive to a perceived lack of care and the erosion of autonomy (Knight et al., 2006; Rogers, 2011).

The young women without children similarly described the importance of relationships in service responses. Stability, routine, and support offered these young women a sense of safety and optimism, but they also often yearned for greater independence. However, their experiences suggested that independent living was not necessarily safe or sustainable. They described being placed in socially isolating accommodation or in contexts that opened them up to social and sexual exploitation and assault. This lack of material safety eroding their emotional and physical safety. When independent living meant living by themselves, they felt particularly unsafe, lonely, and unsupported. Their trauma, loneliness and fears, their sense of not belonging, shaped how they managed the difficulty of independent living, often in unsustainable or dangerous ways.

Among those young women currently unsupported by services, support had been rare or absent throughout their lives. Some had memories of Family Court as the only service response they had experienced, with devastating effects when this resulted in violent fathers being allocated primary care of children. As they grew older, these women sought out their own therapy to understand and heal from their childhood trauma. They either secured stable housing through family or friends or secured private rental on their own—none had received a crisis accommodation service as a result of violence or homelessness. A small number of women reported they received an appropriate crisis response from the police and a specialist DFV agency when they reported violence, and this enabled them to safely move to more permanent housing away from their partner or family.

For the women who did not access supports, housing was a key resource enabling them to build a sense of belonging and familiarity in their lives. Materially safe and sustainable housing allowed them to express a sense of emotional safety, buying possessions and surrounding themselves with mementos helped them create a sense of home. The interviews with this group of women showed that relationships can also be protective factors and are valued by young women. For example, young women in contact with family may find it easier to exit homelessness (Bevitt et al., 2015), and social support and associated self-esteem/self-efficacy are protective factors against future victimization (Tyler et al., 2019).

It is important to summarize learnings from Aboriginal women and women from culturally and linguistically diverse backgrounds regarding what they specifically thought about service support and experiences of safety. The two Aboriginal women with children contacted the DFV service because they had heard positive things about the workers and the service from family and friends. They described not feeling safe physically and emotionally, not only from their partners, but also extended family, and hence wanted a break. The service was able to provide them with this respite but, at the same time, they were able to keep a connection to their extended family, such as visiting them and being able to return to the service. Through this form of cultural safety, young women were able to maintain and strengthen their relationship with family, which was made possible by not living under the same roof (Cripps, 2007).

The younger Aboriginal women without children appreciated having a safe place to stay that had supportive workers around them to navigate practical necessities such as finance, schooling, and social activities. Like the Aboriginal women with children, they too reported appreciating the fact they had somewhere safe to stay but could visit family when they wanted. Maintaining links to family was important to Aboriginal women and enabled them to connect and in some cases mend relationships without having to live with family (Cripps & Habibis, 2019).

The women from culturally and linguistically diverse backgrounds all spoke highly of the service response they received and they described feeling emotionally safe. The quick response from police and crisis DFV services were key to enabling their safety and the alternate accommodation arranged was key to ensuring their stability to recover from the violence and abuse experienced. Two women specifically talked about workers seeking to understand and respond appropriately to their specific cultural needs. Young women experiencing or recovering from the physical experience and continuing trauma of violence and homelessness require time and resource intensity to support longer-term relationship and rapport building (see also Cooper et al., 2009; Gronda, 2009). This also enables young women to understand and recover from trauma.

This study also found that maintaining housing is typically complex because of young women's poverty and, often, reliance on social security payments, and their eagerness to establish new intimate partner relationships while trying to protect and recover from previous violent relationships—both dynamics that contribute to housing instability. For young women, the relationship between DFV and homelessness extends beyond the immediate crisis of leaving a home shared with a perpetrator. They experience ongoing impacts of trauma, which intersect with fear, insecurity, loneliness, and very few supports from family and social networks to encourage the search for a loving relationship. However, without physical, economic, emotional, social, and psychological resources and supports, these relationships may in the medium or longer term contribute to ongoing housing instability or homelessness.

This is not to argue that young women's relationship choices are the primary or direct reason for ongoing housing difficulties. Rather, this article suggests that it is important to attend to the multiple vulnerabilities and complex needs that are created through DFV and/or sexual assault—particularly when such violence occurs over a lifetime. Researchers are clear that individual vulnerabilities must be positioned and responded to in the context of people's biographies (Gaetz et al., 2016) and the structural constraints of poverty (Tually et al., 2009) and the housing market (Blunden & Flanagan, 2022; Warburton et al., 2022). Young women require service responses that are not short-term and crisis-driven but include a consideration of the longer-term processes through which young women can attain safe, appropriate, and sustainable housing and relatedly safe, appropriate, and sustainable relationships.

The implications of this study are that housing support and services for DFV and sexual assault need to prioritize recognition and connection as part of practice. Further attention must be paid to how resources and support are offered in ways that attend to young women's relational needs and their desire to build a future that aligns with their sense of self. Watson and Cuervo (2017) too argue this point, that policies and practices need to focus also on non-material aspects of homelessness through an emphasis on empowerment, self-respect, and autonomy.

The service sector predominantly responds well when it comes to practical and material support, yet responding to young women with complex needs requires more than the material elements of housing. Promoting empowerment, that is, acknowledging young women as people with specific desires and identities and the capacity for autonomy and agency (Flanagan et al., 2019; Watson & Cuervo, 2017) may be an additional and necessary foundation for determining supports. Intergenerational trauma, age, gender, often poverty, and unique forms of oppression stemming from race and ethnicity need to be at the forefront of understanding violence and abuse in young women's lives. In addition, research has shown (Franzway et al., 2019; Rees et al., 2011) that gender-based violence is associated with lifetime mental health disorders. To tackle such complexity a lifetime trauma approach is needed to bring housing and safety policy and support and counseling/therapy practice responses side by side. Thus, exploring how therapeutic services can be embedded as part of the wider service response to help women understand and address their trauma is likely to have positive impacts on their mothering and the development of safe relationships, and on their financial independence and housing autonomy.

## Conclusion

This article has highlighted the interplay of violence, abuse, and homelessness from childhood through to adult intimate partner relationships. This interplay eroded their physical, material, and emotional safety in a process of cumulative trauma and harm. Thus, young women valued service responses that extended beyond the material dimensions of housing and the absence of violence. For the young women, the relational aspects of service provision, and the emotional and cultural safety that arose from these relationships were particularly important in developing a foundation for their futures.
